# Intestinal metaplasia of the renal pelvis: A case report and literature review

**DOI:** 10.3892/ol.2014.2547

**Published:** 2014-09-18

**Authors:** WEIMIN ZHOU, KUANGBIAO ZHONG, JINGRONG WANG, YONGHONG GU, LIHUA HUANG, ZHIQIANG JIANG, LEYE HE

**Affiliations:** 1 Department of Urology, Third Xiangya Hospital, Central South University, Changsha, Hunan 410013, P.R. China; 2 Department of Abdominal Surgery, Jiangxi Cancer Hospital, Nanchang, Jiangxi 330029, P.R. China; 3 Department of Pathology, Third Xiangya Hospital, Central South University, Changsha, Hunan 410013, P.R. China; 4 Center for Medical Experiments, Third Xiangya Hospital, Central South University, Changsha, Hunan 410013, P.R. China

**Keywords:** intestinal metaplasia, renal pelvis, renal calculus, immunohistochemistry

## Abstract

Metaplastic changes in the renal pelvis are infrequent and may be malignant transformations to adenocarcinoma. The current study reports a case of intestinal metaplasia in the right renal pelvis, which was associated with staghorn calculi, in a 56-year-old female. The patient underwent a percutaneous nephrolithotomy. Immunohistochemical assessment of the mucosa of the renal pelvis revealed the positive expression of carcinoembryonic antigen, cytokeratin (CK)-7 and CK20, but negative expression for CK5/6 and vimentin. Furthermore, Ki67 expression was diffusely positive, while p53 was negative. Unlike other previously reported cases, the patient opted for active surveillance as opposed to radical nephrectomy, following the removal of the calculi. No evidence of progression was observed after three years of follow-up. Therefore, etiological treatment and close follow-up may be a suitable treatment option for localized intestinal metaplasia.

## Introduction

The renal pelvis does not contain an intestinal or squamous epithelium, but is normally lined by a urothelium. However, under rare circumstances, particularly those of chronic infection and urinary calculi, the transitional cell epithelium may undergo phenotypical changes, usually in the form of intestinal metaplasia, which are considered to be closely associated with adenocarcinoma (1–5). In addition, for simple intestinal metaplasia of the renal pelvis without abnormal renal function, it has not been well established whether eliminating such stimulating factors may reverse the pathological changes. The present study describes a case of intestinal metaplasia of the renal pelvis where a panel of immunohistochemical biomarkers were applied to aid in the determination of the origin and prognosis of the malignancy. A review of the previously reported cases is also presented. Written informed consent was obtained from the patient.

## Case report

### Patient diagnosis

**Figure 1:**
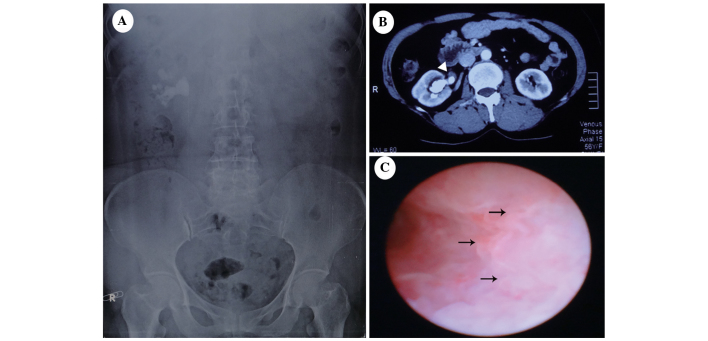
Imaging diagnosis of the patient. (A) Abdominal X-ray revealed right renal staghorn calculi. (B) Contrast enhanced computed tomography demonstrated slight contrast enhancement in the right renal pelvis (white triangle). (C) Mucosal changes under ureteroscopy in the renal pelvis (black arrows).

A 56-year-old female was admitted to the Third Xiangya Hospital of Central South University (Changsha, China) in October 2010 due to backache on the right side that had persisted for one year. The patient had a history of controlled hypertension and recurrent right kidney stones, and therefore, had undergone extracorporeal shock wave lithotripsy on several occasions over ~10 years previously. The physical examination was unremarkable. Abdominal X-ray revealed right renal staghorn calculi (Fig. 1A). Contrast enhanced computerized tomography (CT) demonstrated slight contrast enhancement in a lesion measuring 6×10 mm in diameter in the right renal pelvis (Fig. 1B), indicating that the tumor could not be excised. No enlarged lymph nodes were observed on the CT image. Urine analysis revealed the values of 20 white blood cells per high-power field (normal, <10 white blood cells per high-power field), 35 red blood cells per high-power field (normal, <5 red blood cells per high-power field), proteinuria of 50 mg/l (normal, <100 mg/ml) and no bacteria in urine culture. The serum levels of α-fetoprotein (AFP), carcinoembryonic antigen (CEA) and carbohydrate antigen (CA)19-9 were normal.

### Treatment and follow-up

Percutaneous nephrolithotomy was subsequently performed. Upon nephroscopy, a minor local protrusion, but no evident lump was observed in an area measuring 10×15 mm in the mucosa of the renal pelvis (Fig. 1C). Biopsies were conducted four times for the pathological analysis of the suspicious lesions. Following the removal of the calculi, the recovery was uneventful, and the patient was discharged from the hospital six days later. The pathological diagnosis was of significant intestinal metaplasia, and radical nephrectomy or local lesions electrovaporization was subsequently recommended, however the patient selected active surveillance. To date, subsequent to three years of follow-up, the patient is alive, with no evidence of tumor progression observed on CT scan and with normal serum AFP, CEA and CA19-9 levels.

### Pathological findings

**Figure 2:**
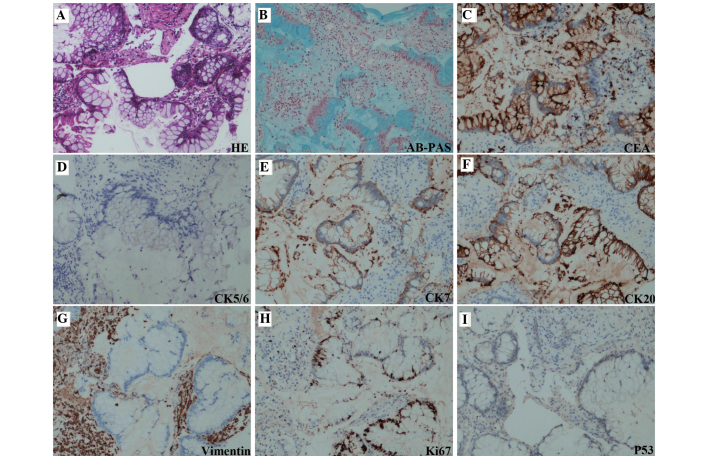
Histological features of the renal pelvis. (A) Hematoxylin-eosin staining revealing significant intestinal metaplasia and inflammation infiltration; part of the glandular epithelium showed low-grade dysplasia. (B) Alcian blue-periodic acid Schiff (AB-PAS) staining revealing abundant mucin within the cytoplasm of the glandular epithelium. Immunohistochemical staining showing (C) the positive expression of carcinoembryonic antigen; (D) CK5/6-negative expression; (E and F) the positive expression of CK7 (weak) and CK20 (strong); (G) vimentin-negative expression; (H) the diffusely-positive nuclear staining of Ki67; and (I) p53-negative expression. Original magnification, ×200. CK, cytokeratin.

Hematoxylin-eosin (HE) staining revealed that the biopsied tissues were composed of significant intestinal metaplasia with abundant goblet cells (Fig. 2A). Part of the glandular epithelium exhibited low-grade dysplasia. No squamous metaplasia or invasive adenocarcinoma was observed. In addition to the glandular zone, infiltration with lymphocytes and plasma cells was observed. Staining with alcian blue-periodic acid Schiff revealed abundant mucin within the cytoplasm of the glandular epithelium (Fig. 2B). In all the biopsies, no normal transitional epithelium was observed. Immunohistochemical analysis revealed positive expression for CEA, CK7 and CK20, however, negative expression was observed for CK5/6 and vimentin in the metaplastic urothelium (Fig. 2C–G). Furthermore, Ki67 expression was diffusely positive with an mean labeling index of 12% in 10 random microscopic fields, whereas p53 was negative in all instances (Fig. 2H and I).

## Discussion

Cases of metaplasia of the renal pelvis without associated malignancy are extremely rare, and to the best of our knowledge, only 18 cases have been previously reported in the English language literature (Table I) (1,6–17). A review of this literature showed that the mean age of these patients was 51.2±14.0 years and the male to female ratio was 2.6:1, with a male predominance. In total, eight of the 18 subjects exhibited intestinal metaplasia (single intestinal or combined with squamous metaplasia). Almost all of the reported subjects had experienced a long history of irritations of the renal pelvis, the majority of which were chronic urinary tract infections, hydronephrosis and calculus. Of the reported subjects, 15 patients (83.3%) exhibited calculi and more than half of the subjects suffered from a large calculus or multiple calculi of the renal pelvis (11/18; 61.1%). Therefore, the long-term effects of chronic irritations are likely to be associated with metaplasia of the urothelium of the renal pelvis.

The exact mechanism by which intestinal metaplasia occurs is not entirely understood, however, it has been hypothesized to be associated with the endodermal origin of the embryonal cloaca and intestine (12). Therefore, the divergent metaplastic potentialities of the urinary transitional epithelium to squamous, mucinous or intestinal metaplasia can be readily explained (12). In addition, intestinal metaplasia within the upper urinary tract is extremely unusual and may prompt the surgeon to consider a primary intestinal pathological cause (13). Usually, a normal urothelium expresses simple epithelial cytokeratins, including CK5/6, CK7 and CK20, while in gastroenteric tumors, this does not occur (1,21). In the present case, CK5/6 expression was absent in the metaplastic epithelium, however, stronger CK20 expression was present. Therefore, we proposed that the renal pelvis epithelium underwent changes in phenotype in the intestinal metaplasia and may not be of primary intestinal pathology.

Precancerous changes are hypothesized to contribute to the metaplastic changes in the urothelium, and adenocarcinomas may arise from metaplastic changes of an epithelium that is potentially unstable (18). The presence of adenocarcinoma in combination with intestinal metaplasia has been frequently observed (3–5,19,20). Similarly, with intestinal metaplasia of the renal pelvis, a significant history of chronic irritations, including inflammation and calculi, are also present in the majority of cases of adenocarcinoma (2–4,19). Spires *et al* (2) reviewed a total of 59 cases of adenocarcinoma and observed that tubulovillous and mucinous tissue types, which accounted for 93% of cases, were morphologically similar to intestinal tumors, and therefore may arise from foci of intestinal metaplasia. Considering this, adenocarcinomas are likely to develop from the progressive transformation of these metaplastic cells in a stepwise adenoma-carcinoma sequence, possibly in a similar manner to colonic carcinogenesis (3). Notably, these conclusions were predominantly based on the synchronous presence of cancer in the specimen, which also contained metaplastic changes. In addition, in numerous other cases, the neoplasms arose without any preceding metaplastic changes (21,22). Therefore, the malignancy of metaplasia in the renal pelvis remains controversial.

The occurrence of metaplasia in the renal pelvis without an associated malignancy is rare, as reviewed previously (16). In the bladder, cystitis glandularis and intestinal metaplasia have been proposed to represent precursors of bladder adenocarcinoma (23,24). However, this notion has now been challenged. By observing 53 patients for 10 years and 136 patients for 2.6 years, respectively, Corica *et al* (25) and Smith *et al* (24) revealed that cystitis glandularis or intestinal metaplasia had no tendency towards carcinoma. The identification and removal of the causes of cystitis glandularis, such as upper urinary tract obstruction, were considered as the most important management methods (26). However, in the renal pelvis, further evaluation ir required to determine whether intestinal metaplasia has the potential to progress to adenocarcinoma and whether etiological treatment may reverse the pathological changes. To date, no reports have traced progression to malignancy in patients with a final diagnosis of intestinal metaplasia of the renal pelvis.

Elevated serum levels of AFP, CEA and CA19-9 have been reported in several studies of adenocarcinoma of the renal pelvis and were considered to be effective prognostic biomarkers (22,27). In the current case, the serum levels of AFP, CEA and CA19-9 were normal and remained normal subsequent to three years of follow-up. However, immunohistochemical staining revealed strong CEA expression. In addition, the expression of tumor markers p53 and Ki67 was also evaluated in the tissue. The results revealed p53-negative expression, but diffusely-positive expression for Ki67, suggesting a potential proliferation ability of the intestinal metaplasia of the renal pelvis. Notably, in this case, the biopsies that were obtained were superficial to a certain extent, and it is possible that the potential adenocarcinoma below the mucosa may have been overlooked, as the CT scan suggested limited lesions in the renal pelvis. Due to the poor prognosis of adenocarcinoma of the renal pelvis, for which the majority of patients succumb to the disease within two to five years (27), radical nephrectomy or local lesions electrovaporization remained the recommended treatment for the current patient following the removal of the calculi by percutaneous nephrolithotomy; however, the patient selected active surveillance. Subsequent to three years of follow-up by CT imaging every six months, no further progression was observed. The surveillance will continue for the foreseeable future.

In conclusion, intestinal metaplasia of the renal pelvis is closely associated with chronic stimuli, particularly from complex calculi and urinary tract infections. It remains controversial whether intestinal metaplasia may progress to adenocarcinoma. Etiological treatment and close follow-up may be suitable and practical for local intestinal metaplasia, however this option may pose an increased risk. Future studies examining the long-term outcomes in a larger series of patients with intestinal metaplasia of the renal pelvis may be of value to further delineate the association between intestinal metaplasia and renal pelvis carcinoma.

## Figures and Tables

**Table I:** Reports of metaplasia of the renal pelvis without associated malignancy.

First author/s, year (ref.)	Gender/age, years	Type of metaplasia	Calculus	Associated condition
Foot, 1944 (6)	F/54	Intestinal	++	Pyonephrosis
Torassa, 1948 (7)	M/46	Intestinal	++	Pyonephrosis and multiple abscesses
Maclean and Fowler, 1956 (8)	F/39	Intestinal	++	Chronic pyelonephritis
Krag and Alcott, 1957 (9)	M/52	Intestinal and squamous	++	Hydronephrosis and pyonephrosis
Gordan, 1963 (10)	M/55	Intestinal and squamous	+	Chronic pyelonephritis
	M/50	Glandular	−	Bladder dysfunction and pyonephrosis
Towers, 1963 (11)	F/55	Cystic and intestinal	U	Pyonephrosis
Salm, 1969 (12)	M/55	Intestinal and squamous	++	Phronic pyelonephritis
Ward, 1971 (13)	M/49	Glandular and squamous	++	Pyonephrosis.
	M/40	Glandular	+	U
	M/64	Squamous and glandular	+	Pyonephrosis
Blacklock *et al*, 1983 (14)	M/61	Squamous and mucinous	++	Chronic pyelonephritis
	M/21	Squamous and mucinous	++	Chronic pyelonephritis and hydronephrosis
Lam and Choi, 1995 (15)	M/79	Mucinous	++	Chronic pyelonephritis and nephrosclerosis
Mathur *et al*, 2004 (16)	M/40	Mucinous	++	Chronic pyelonephritis
Deniz *et al*, 2010 (1)	M/32	Intestinal	+	Chronic pyelonephritis and renal atrophy
Siderits *et al*, 2012 (17)	F/74	Squamous	−	Renal obstruction and pyohydronephrosis
Present case	F/56	Intestinal	++	Pyonephrosis

+, simple calculus; ++, large calculus or multiple calculi; −, no history of calculus; U, unknown; F, female; M, male.
